# Diagnostic accuracy of multiparametric ultrasound in the diagnosis of prostate cancer: systematic review and meta-analysis

**DOI:** 10.1186/s13244-023-01543-1

**Published:** 2023-11-24

**Authors:** Yun Tang, Xingsheng Li, Qing Jiang, Lingyun Zhai

**Affiliations:** 1 Department of Geriatric Medicine, The Second Affiliated Hospital of Chongqing Medical University, Chongqing, 400010 China; 2 Longmen Hao Street Community Health Service Center, Nan’an District, Chongqing, 401336 China; 3 Department of Urology, The Second Affiliated Hospital of Chongqing Medical University, Chongqing, 400010 China

**Keywords:** Prostate cancer, Multiparametric ultrasound, Contrast-enhanced, Elastography

## Abstract

**Objectives:**

Ultrasound (US) technology has recently made advances that have led to the development of modalities including elastography and contrast-enhanced ultrasound. The use of different US modalities in combination may increase the accuracy of PCa diagnosis. This study aims to assess the diagnostic accuracy of multiparametric ultrasound (mpUS) in the PCa diagnosis.

**Methods:**

Through September 2023, we searched through Cochrane CENTRAL, PubMed, Embase, Scopus, Web of Science, ClinicalTrial.gov, and Google Scholar for relevant studies. We used standard methods recommended for meta-analyses of diagnostic evaluation. We plot the SROC curve, which stands for summary receiver operating characteristic. To determine how confounding factors affected the results, meta-regression analysis was used.

**Results:**

Finally, 1004 patients from 8 studies that were included in this research were examined. The diagnostic odds ratio for PCa was 20 (95% confidence interval (CI), 8–49) and the pooled estimates of mpUS for diagnosis were as follows: sensitivity, 0.88 (95% CI, 0.81–0.93); specificity, 0.72 (95% CI, 0.59–0.83); positive predictive value, 0.75 (95% CI, 0.63–0.87); and negative predictive value, 0.82 (95% CI, 0.71–0.93). The area under the SROC curve was 0.89 (95% CI, 0.86–0.92). There was a significant heterogeneity among the studies (*p* < 0.01). According to meta-regression, both the sensitivity and specificity of mpUS in the diagnosis of clinically significant PCa (csPCa) were inferior to any PCa.

**Conclusion:**

The diagnostic accuracy of mpUS in the diagnosis of PCa is moderate, but the accuracy in the diagnosis of csPCa is significantly lower than any PCa. More relevant research is needed in the future.

**Critical relevance statement:**

This study provides urologists and sonographers with useful data by summarizing the accuracy of multiparametric ultrasound in the detection of prostate cancer.

**Key points:**

• Recent studies focused on the role of multiparametric ultrasound in the diagnosis of prostate cancer.

• This meta-analysis revealed that multiparametric ultrasound has moderate diagnostic accuracy for prostate cancer.

• The diagnostic accuracy of multiparametric ultrasound in the diagnosis of clinically significant prostate cancer is significantly lower than any prostate cancer.

**Graphical Abstract:**

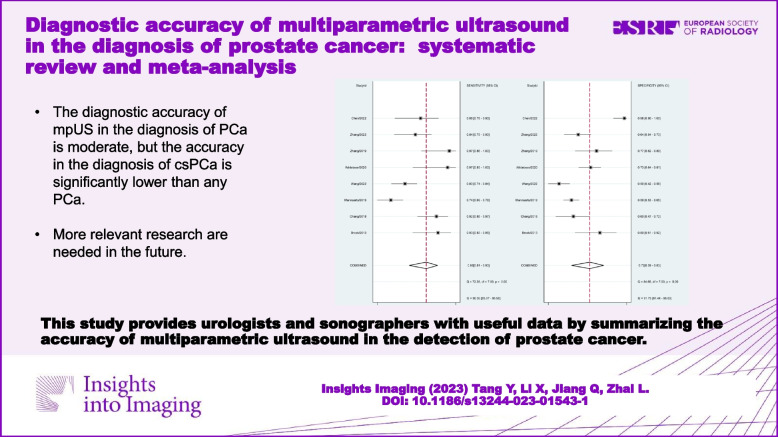

**Supplementary Information:**

The online version contains supplementary material available at 10.1186/s13244-023-01543-1.

## Introduction

Prostate cancer is the fourth most commonly diagnosed cancer in the world and the second leading cause of cancer death in men in the USA (https://www.cancer.net/cancer-types/prostate-cancer/statistics). Transrectal ultrasound-guided prostate biopsy has long been the gold standard for diagnosing prostate cancer. However, compared to prostate ultrasound alone, prostate multiparametric magnetic resonance imaging (mpMRI) plays an increasingly important role in the diagnosis of PCa in the current clinical practice, especially for MRI-targeted biopsy or MRI/ultrasound fusion targeted biopsy [1]. Compared with MRI, ultrasound has advantages of convenience, good economy, and not restricted by claustrophobia, and it has natural advantages in guiding biopsy and local treatment [2, 3].

In recent years, new ultrasound techniques, such as contrast-enhanced ultrasound and elastic imaging techniques, have been increasingly reported and improved the accuracy of traditional ultrasound in the diagnosis of prostate cancer [4–6]. Similar to multiparametric MRI, some researchers have reported that the combination of multiple ultrasound techniques may have certain advantages over a single ultrasound technique [7, 8]. Multi-parametric prostate ultrasound is a non-broadly used albeit potentially valuable diagnostic approach with an estimated high benefit/cost ratio. However, the diagnostic accuracy of multi-parameter ultrasound for prostate cancer is still unclear. The purpose of this study was to investigate the diagnostic performance of mpUS for prostate cancer. According to the ultrasound techniques used in the existing research papers, we define multiparametric ultrasound as ultrasound that combines three or more kinds of modalities and at least includes contrast-enhanced ultrasound and elastic imaging techniques.

## Materials and methods

### Search methods and selection standards

This meta-analysis was registered in PROSPERO (CRD42023463673). Multiparametric ultrasound is defined as ultrasound that combines three or more kinds of modalities and at least includes contrast-enhanced ultrasound and elastic imaging techniques. Therefore, our overall search strategies are #1 contrast-enhanced, #2 elastography, #3 multiparametric ultrasound, and #4 prostate cancer. We combined [(#1 and #2) or #3] and #4. All possible synonyms were used. The detailed search strategy was displayed in the Supplementary material.

We looked through the Cochrane CENTRAL, PubMed, Embase, Scopus, Web of Science, ClinicalTrial.gov, and Google Scholar between the database’s establishment and September 2023. Only English-language articles are featured.

Studies were considered if they satisfied all of the following requirements: (1) patients undergoing radical prostatectomy or prostate biopsy made up the research population, (2) multiparametric ultrasound was performed prior to biopsy or surgery, (3) pathology was the reference standard, and (4) relevant data can be accurately extracted.

Studies were disqualified if they fell under one of the following categories: (1) review or meta-analysis in the paper; (2) extremely overlapping reported populations; (3) abstract-only papers, conferences, or books; and (4) papers containing histo-scanning only or micro-US only.

### Data gathering and quality evaluation

After the database searches, all titles and abstracts were checked by two authors separately. If either author considers the article eligible, both authors will read the full article to determine the inclusion in the study. Then, data was extracted from the included studies by the two authors independently. Consensus is to be used to resolve disagreements. Studies that did not fit all the requirements for inclusion were rejected. The quality of the included studies was assessed using the Diagnostic Accuracy Research Quality Assessment (QUADAS-2) [9]. Two authors individually assessed each study. The differences shall be settled through discussion, and if there are still differences, they shall be settled through third-party arbitration.

### Statistical analysis

We used established methodologies to conduct this diagnostic meta-analysis, as stated in the PRISMA statement [10]. All of the studies’ true-positive, false-negative, false-positive, and true-negative values were calculated. Positive likelihood ratio, negative likelihood ratio, sensitivity, specificity, diagnostic odds ratio, and 95% confidence interval (CI) were computed. We created the summary receiver operating characteristic (SROC) curve and plotted the forest plots. A random effects meta-analysis was used to generate the pooled positive and negative predictive values, as well as their respective 95% confidence intervals.

The *I*^2^ approach was used to calculate statistical heterogeneity [11]. To evaluate the potential sources of heterogeneity, a meta-regression was conducted. The bivariable mixed-effect regression model was employed [12]. Deeks’ funnel plot was used to identify potential publishing bias [13]. *p* < 0.05 was regarded as statistically significant. Revman 5.3 and the MIDAS module of STATA 14.0 were used for this meta-analysis (https://econpapers.repec.org/paper/bocasug07/4.htm) [14].

## Results

### Eligible research and evaluation of quality

**Fig. 1:**
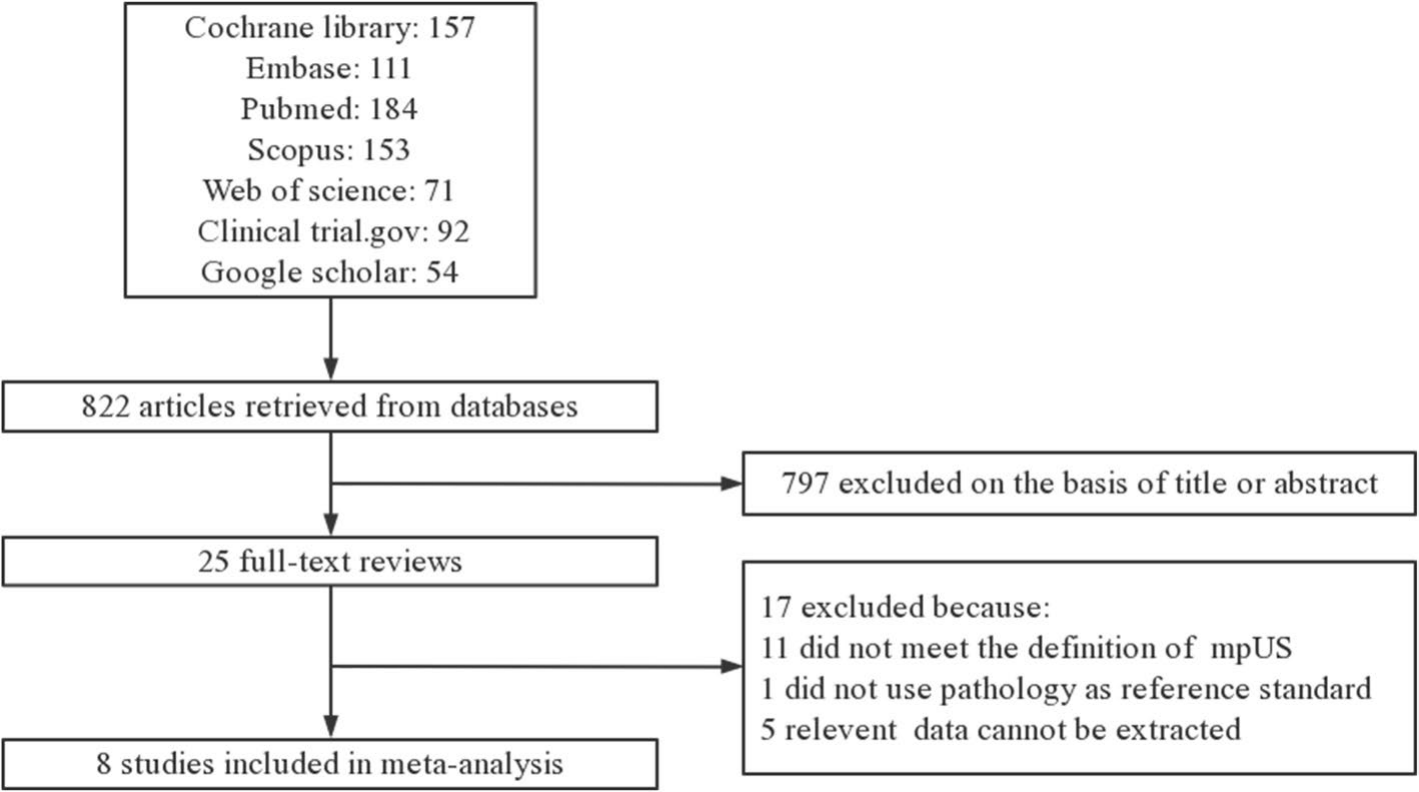
Study selection

After searching, 822 literatures were preliminarily included. After reviewing the titles and abstracts, 25 literatures were selected. A total of eight studies were included in this meta-analysis after a full-text review (Fig. 1) [15–22].

The eight articles included a total of 1004 patients. The number of patients in these studies ranged from 48 to 315. Some of the researchers analyzed different areas of the prostate separately, so a total of 1783 issues were included in the analysis. All eight studies were single-center studies, five of which used a prospective design. There were five studies containing patients who received prostate biopsy because of an elevated prostate-specific antigen, and the reference standard was biopsy pathology. In the other three studies, biopsy-proven prostate cancer patients underwent radical prostatectomy, and the reference standard was radical prostatectomy pathology. All patients underwent multiparametric ultrasonography before biopsy or surgery. The ultrasound modalities used in all eight studies included B-mode, elastography, and contrast-enhanced ultrasound. Elastic imaging techniques varied between studies, with shear-wave elastography used in 3 studies and strain elastography used in 5 studies. Pathological positivity in 4 studies was defined as any PCa, with the other 4 studies defined as csPCa. The definition of csPCa varied between these studies. Table 1 displays the key research characteristics.

**Table 1:** Summary of included studies

First author and year	Study design	Imaging modality	Number of patients	Patient characteristics	Outcome	Reference standard	Elastography technique
Brock 2013 [15]	Single-center prospective	B-mode + elastography + CEUS	86	Biopsy-proven prostate cancer	Any PCa	RP histology	Strain elastography
Chang 2018 [16]	Single-center prospective	B-mode + elastography + CEUS	153	PSA ≥ 4.0 ng/mL	Any PCa	10-core SBx + 2-core TBx	Strain elastography
Mannaerts 2019 [17]	Single-center prospective	B-mode + elastography + CEUS	48	Biopsy-proven prostate cancer	GS ≥ 3 + 4 = 7, tumor volume ≥ 0.5 mL, EPE or pN1	RP histology	Shear-wave elastography
Wang 2022 [18]	Single-center retrospective	B-mode + elastography + CEUS	315	Symptomatic; ≥ 40 years; PSA ≥ 4.0 ng/mL; abnormal DRE	GS ≥ 3 + 4	TBx	Strain elastography
Wildeboer 2020 [19]	Single-center prospective	B-mode + elastography + CEUS	48	Biopsy-confirmed PCa; PSA ≤ 20 ng/mL; PV < 80 mL; no EPE	GS > 3 + 4	RP histology	Shear-wave elastography
Zhang 2019 [20]	Single-center prospective	Grayscale and color Doppler + elastography + CEUS	78	PSA > 4.0 ng/mL or increasing PSA or abnormal DRE	Any PCa	12-core SBx	Shear-wave elastography
Zhang 2022 [21]	Single-center retrospective	B-mode + elastography + CEUS	160	Biopsy-naive men with PSA > 4 ng/mL	GS ≥ 3 + 4	12-core SBx + TBx	Strain elastography
Chen 2022 [22]	Single-center retrospective	Grayscale and color Doppler + elastography + CEUS	116	PSA > 4.0 ng/mL	Any PCa	SBx + 2-core TBx	Strain elastography

*Abbreviations*: *CEUS* contrast-enhanced ultrasonography, *DRE* digital rectal examination, *EPE* extraprostatic extension, *GS* Gleason score, *PCa* prostate cancer, *PSA* prostate specific antigen, *PV* prostate volume, *RP* radical prostatectomy, *SBx* systematic biopsy, *TBx* targeted biopsy

**Fig. 2:**
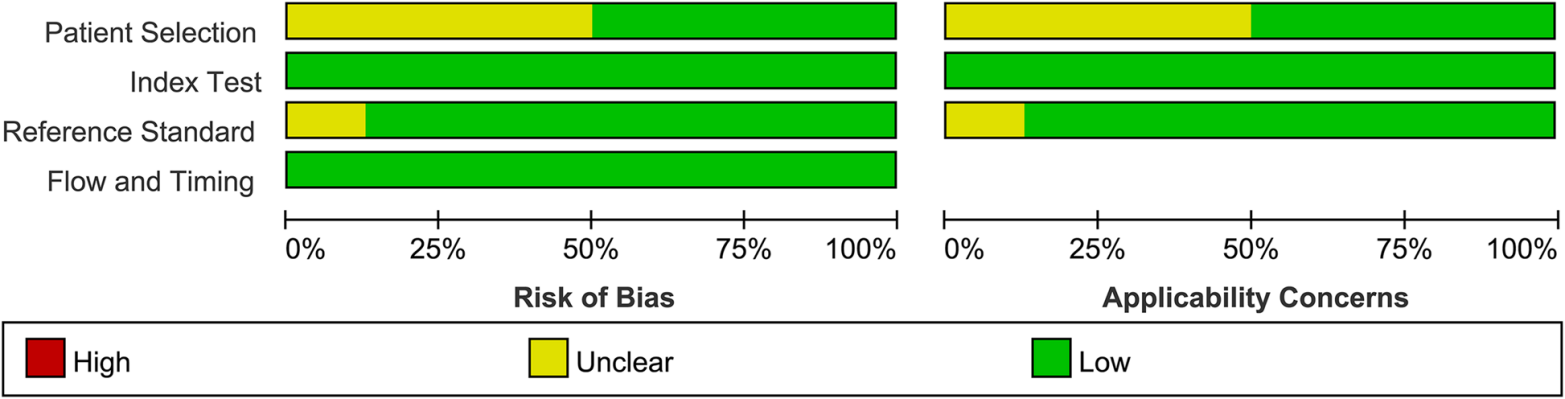
Graph demonstrating the listed studies’ quality assessment

**Fig. 3:**
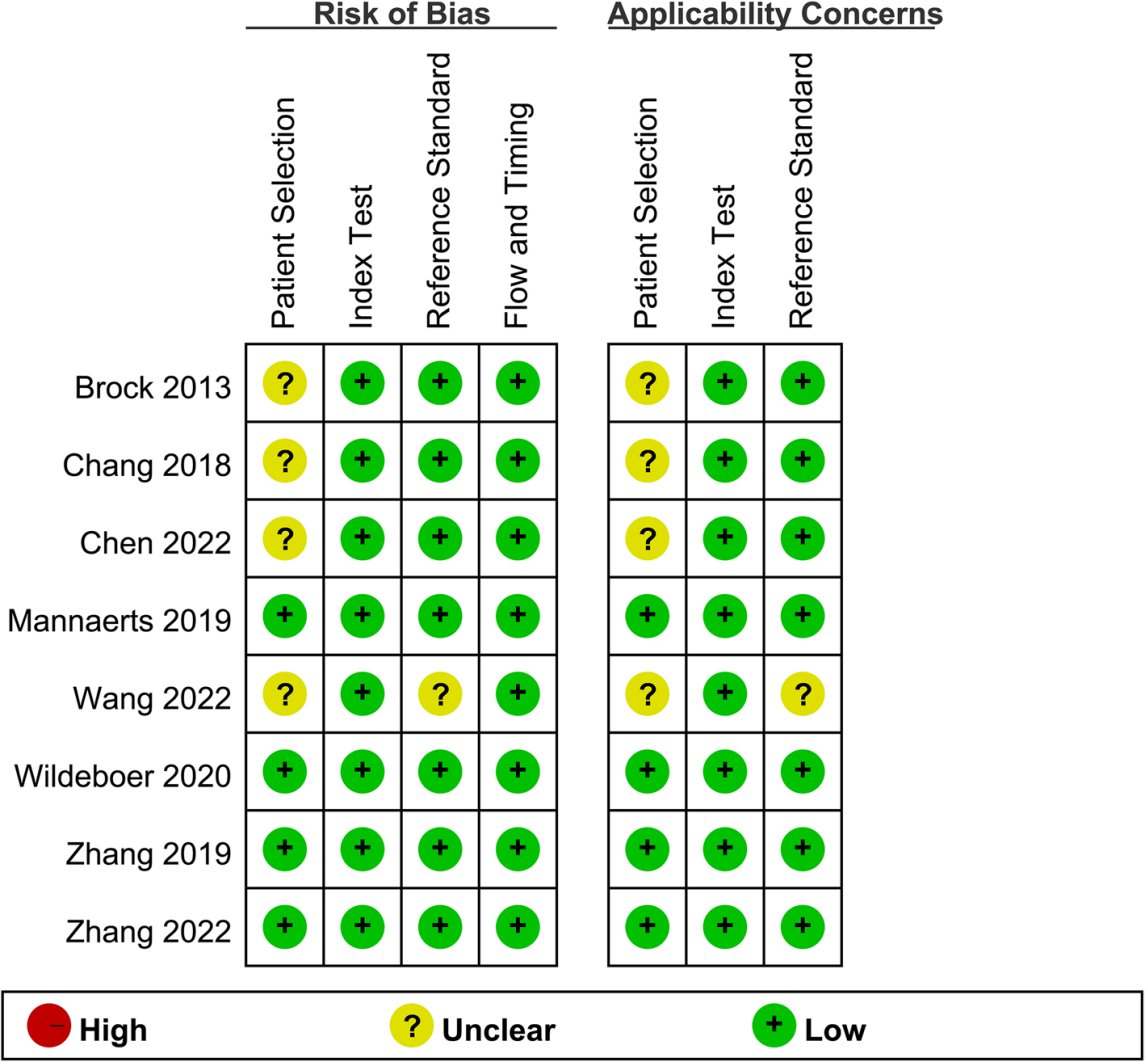
Chart showing the listed studies’ quality assessment

In Figs. 2 and 3, the QUANAS-2 quality data are summarized. Because there were only low to uncertain bias risk and applicability concerns, we did not remove any papers from the analysis.

### Diagnostic accuracy

**Fig. 4:**
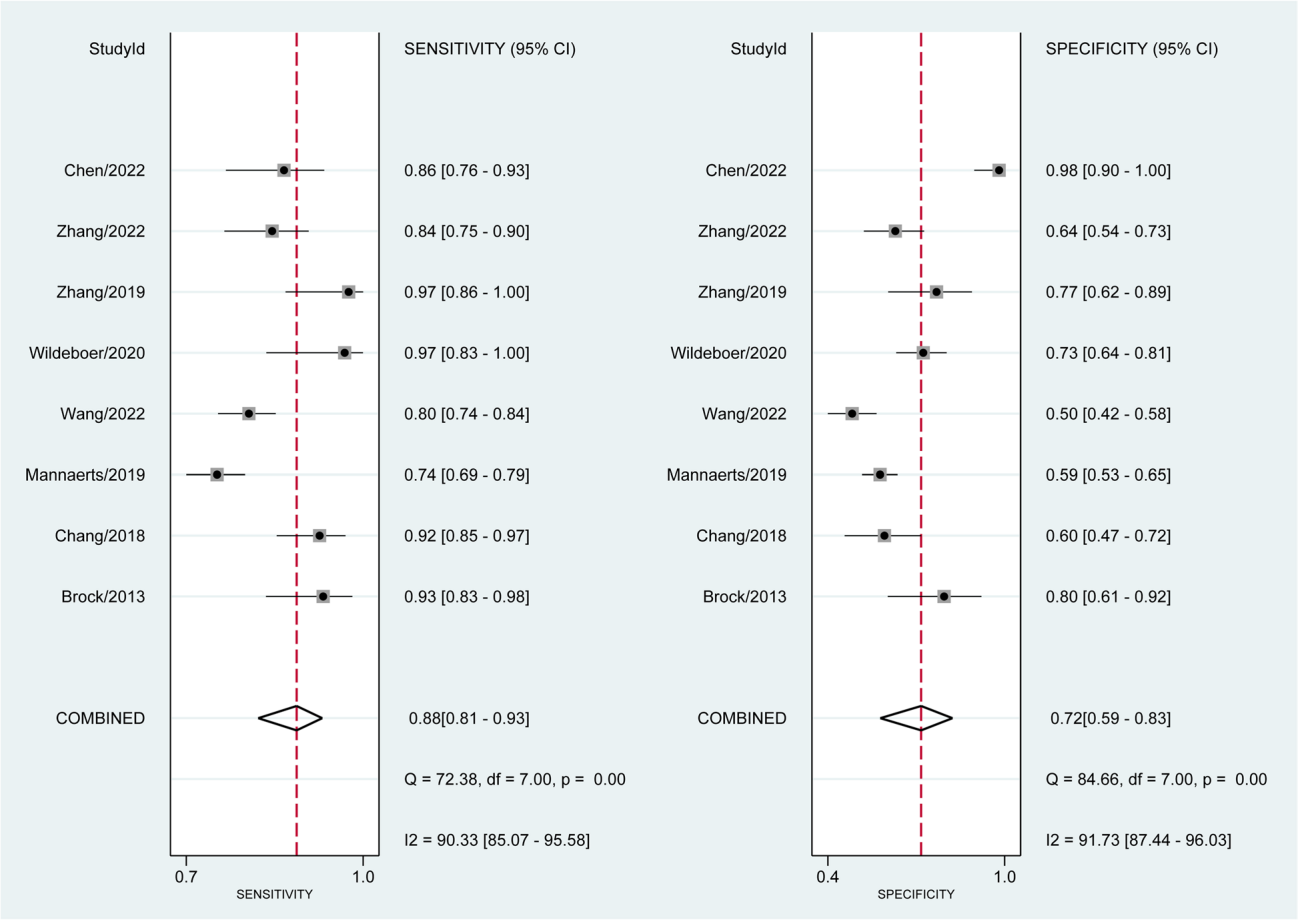
Sensitivity and specificity forest plots

**Fig. 5:**
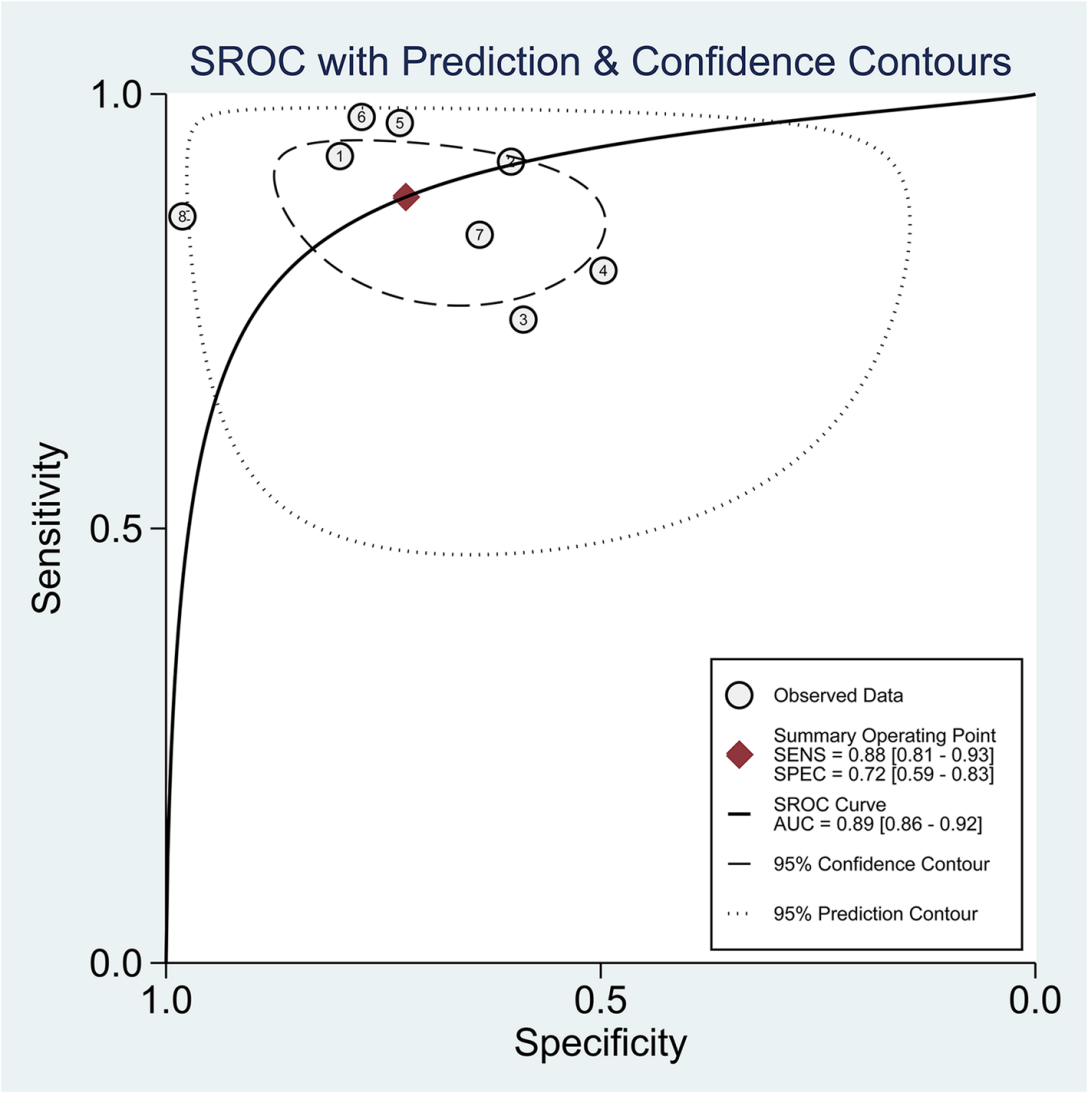
Summary ROC curve

The forest plot in Fig. 4 showed the sensitivity and specificity of each research. The estimated pooled mpUS sensitivity, specificity, positive predictive value, and negative predictive value for the diagnosis of prostate cancer were 0.88 (95% CI, 0.81–0.93), 0.72 (95% CI, 0.59–0.83), 0.75 (95% CI, 0.63–0.87), and 0.82 (95% CI, 0.71–0.93). The diagnostic odds ratio was 20 (95% CI, 8–49), and the positive likelihood ratio was 3.2 (95% CI, 2.0–5.1). The negative likelihood ratio was 0.16 (95% CI, 0.10–0.28). Figure 5 shows the SROC curve, which displayed an area under the curve of 0.89 (95% CI, 0.86–0.92). The diagnostic estimates for each study were displayed in Table 2.

**Table 2:** Diagnostic estimates of each study

First author and year	Diagnostic estimates (95%CI)								
	TP	FP	FN	TN	PLR	NLR	DOR	Sensitivity	Specificity
Brock 2013 [15]	52	6	4	24	4.64 [2.26–9.53]	0.09 [0.03–0.23]	52.00 [13.42–201.48]	0.93 [0.83–0.98]	0.80 [0.61–0.92]
Chang 2018 [16]	83	25	7	38	2.32 [1.70–3.17]	0.13 [0.06–0.27]	18.02 [7.17–45.31]	0.92 [0.85–0.97]	0.60 [0.47–0.72]
Mannaerts 2019 [17]	214	118	75	169	1.80 [1.54–2.10]	0.44 [0.35–0.55]	4.09 [2.87–5.82]	0.74 [0.69–0.79]	0.59 [0.53–0.65]
Wang 2022 [18]	204	80	52	79	1.58 [1.34–1.87]	0.41 [0.31–0.55]	3.87 [2.51–5.99]	0.80 [0.74–0.84]	0.50 [0.42–0.58]
Wildeboer 2020 [19]	29	32	1	87	3.59 [2.65–4.87]	0.05 [0.01–0.31]	78.84 [10.31–602.87]	0.97 [0.83–1.00]	0.73 [0.64–0.81]
Zhang 2019 [20]	37	9	1	31	4.33 [2.43–7.71]	0.03 [0.00–0.24]	127.44 [15.29–1062.17]	0.97 [0.86–1.00]	0.77 [0.62–0.89]
Zhang 2022 [21]	88	35	17	62	2.32 [1.76–3.07]	0.25 [0.16–0.40]	9.17 [4.72–17.82]	0.84 [0.75–0.90]	0.64 [0.54–0.73]
Chen 2022 [22]	61	1	10	52	45.54 [6.52–318.03]	0.14 [0.08–0.26]	317.20 [39.29–2561.07]	0.86 [0.76–0.93]	0.98 [0.90–1.00]

*Abbreviations*: *CI* confidence interval, *DOR* diagnostic odds ratio, *FN* false negative, *FP* false positive, *NLR* negative likelihood ratio, *PLR* positive likelihood ratio, *TN* true negative, *TP* true positive

### Publication bias and heterogeneity

The studies had high levels of heterogeneity in terms of their sensitivity (*p* < 0.01, *I*^2^ = 90.33%) and specificity (*p* < 0.01, *I*^2^ = 91.73%). As potential sources of heterogeneity, we took into account research design, various elastography techniques, the definition of PCa, and the reference standard. The following standards were used in a subgroup analysis: (1) two categories of study designs were used—three retrospective studies and five prospective studies; (2) in terms of the elastography technique, there were two groups—three studies using shear-wave elastography and five studies using strain elastography; (3) three studies using radical prostatectomy pathology as the reference standard and five studies using biopsy pathology; and (4) four studies using csPCa and four studies using any PCa as the definition of PCa.

According to the meta-regression (Table 3), the PCa definition was the main cause of the heterogeneity in sensitivity and specificity: both the sensitivity (0.81 vs 0.92, *p* < 0.01) and specificity (0.62 vs 0.82, *p* = 0.02) of diagnosing csPCa were inferior to any PCa. The other potential sources such as study design, types of elastography technique, and reference standards did not contribute to the heterogeneity.

**Table 3:** Meta-regression and subgroup analysis of the included studies

Variables	Subgroup	Number of studies	Sensitivity, 95% CI	*p*	Specificity, 95% CI	*p*
Study design	Prospective	5	0.91 [0.85–0.97]	0.38	0.71 [0.55–0.87]	0.41
	Retrospective	3	0.84 [0.74–0.94]		0.75 [0.56–0.94]	
Elastography technique	SWE	3	0.90 [0.80–0.99]	0.30	0.71 [0.51–0.90]	0.45
	SE	5	0.88 [0.81–0.94]		0.74 [0.58–0.89]	
Reference standard	RP	3	0.88 [0.79–0.97]	0.10	0.71 [0.52–0.91]	0.50
	Biopsy	5	0.88 [0.82–0.95]		0.73 [0.58–0.88]	
Definition of PCa	csPCa	4	0.81 [0.74–0.88]	**< 0.01**	0.62 [0.46–0.77]	**0.02**
	Any PCa	4	0.92 [0.88–0.96]		0.82 [0.70–0.93]	

*Abbreviations*: *CI* confidence interval, *csPCa* clinically significant PCa, *PCa* prostate cancer, *RP* radical prostatectomy, *SE* strain elastography, *SWE* shear-wave elastography

**Fig. 6:**
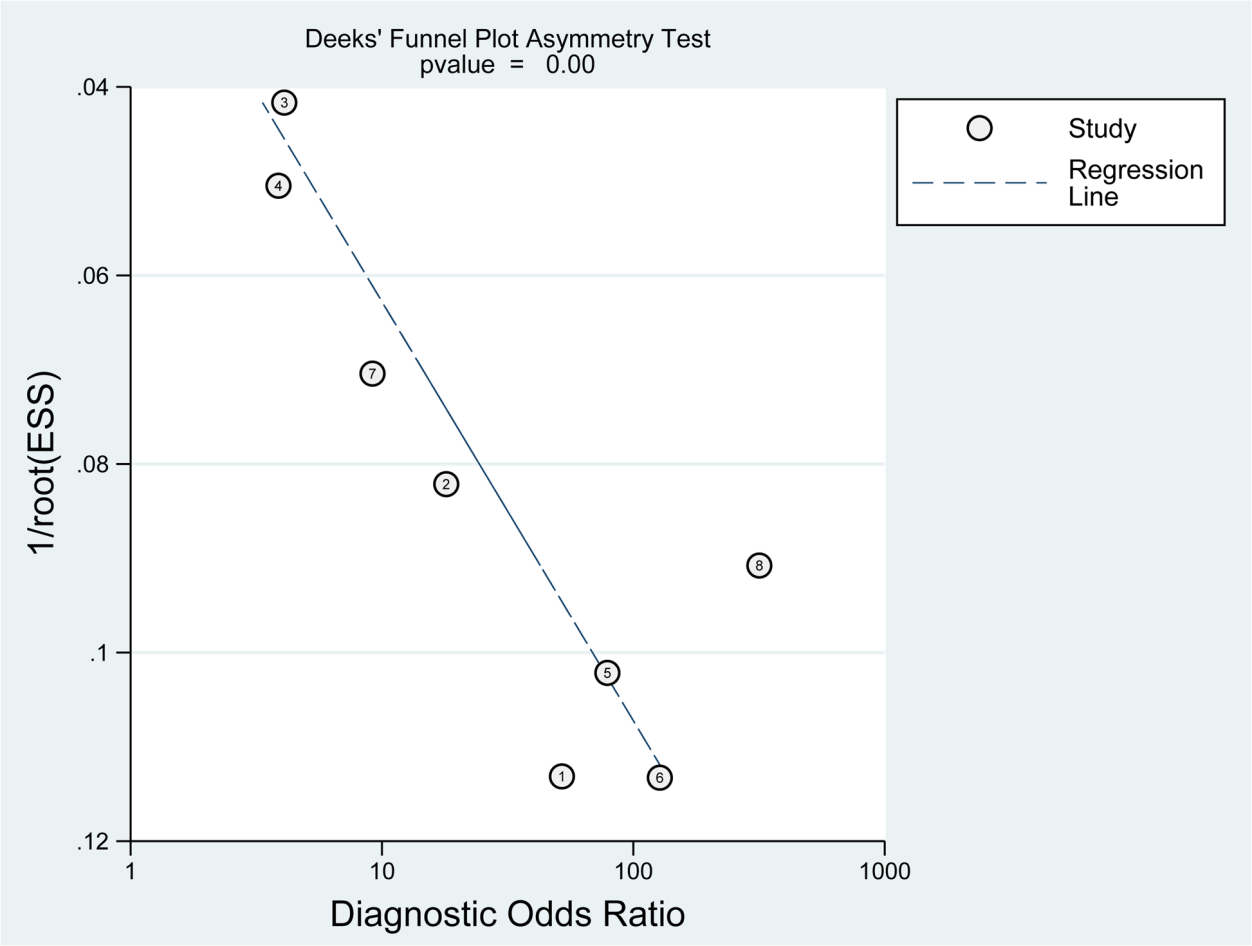
Deeks’ publishing bias funnel plots

Figure 6 shows the results of Deeks’ funnel plot asymmetry test, which revealed publication bias. The bias coefficient was significant (*p* < 0.01) and stood at 51.82.

## Discussion

In the past few years, imaging diagnosis of PCa has mainly relied on mpMRI, and mpMRI with prostate imaging reporting and data system have indeed demonstrated good diagnostic performance, especially for clinically significant prostate cancer [23]. However, there are some evident benefits of US technology, such as lower prices, real-time scans, suitability for those who are MRI contraindicated, and accessibility in the office setting, which plays an irreplaceable role in clinical practice today [2].

New US modalities have been established as a result of recent improvements in ultrasound technology. Contrast-enhanced ultrasound can show angiogenesis more clearly and improve the chances of detection of malignant tumors [24]. Previous studies have explored the value of contrast-enhanced ultrasound in the diagnosis of prostate cancer, with a 91.7% positive predictive value, a 79.3% sensitivity, and an 83.7% accuracy. Contrast-enhanced ultrasound-guided targeted biopsy may dramatically increase the rate of cancer detection compared to a 12-core systematic biopsy [25–28]. Elastography can measure and quantify the hardness of tissue. Elastography is also used to diagnose prostate cancer because malignant tissue is often harder than benign tissue [29]. In a meta-analysis of 508 patients, the sensitivity and specificity for PCa detection were 72% and 76%, respectively, when strain elastography and histology were compared after radical prostatectomy [30]. When biopsy pathology was used as a reference, another meta-analysis of shear-wave elastography with 16 studies indicated that it had 85% sensitivity and 85% specificity for the detection of prostate cancer. When the pathology following radical surgery was employed as the reference standard, the sensitivity and specificity were respectively 71% and 74% [31].

As was already mentioned, new ultrasound techniques have produced encouraging results. However, it is unclear if a single novel US modality can provide enough findings for the diagnosis of prostate cancer. MpUS and mpMRI share a similar idea. Given the success of mpMRI in PCa, it is also possible to combine multiple ultrasound modalities to achieve more reliable performance.

Because of the characteristics of ultrasound, mpUS may be more advantageous in guiding targeted biopsy and focal therapy.

It is our understanding that this is the first meta-analysis focused on the role of mpUS in the diagnosis of prostate cancer, which provides valuable information to urologists and sonographer. We found that mpUS had moderate accuracy in prostate cancer diagnosis. Our aim as uro-radiologists is to predict clinically significant prostate cancer. It seems that the sensitivity and specificity of mpUS in the diagnosis of clinically significant prostate cancer are lower than that in the diagnosis of any prostate cancer as the result of meta-regression, which is contrary to the characteristics of mpMRI [32]. However, only four studies assessed the diagnosis of csPCa in this meta-analysis, and the definitions of csPCa varied between these studies. The diagnostic performance of mpUS for csPCa needs further investigation.

Other potential sources of heterogeneity were not found in the meta-regression. With regard to the study design, although the authors of several articles claimed that their research is prospective, whether the “prospective” studies kept a prospective design was not clear. As to the elastography technique, we found no significant differences between strain and SWE techniques. Consistent with our results, both methods have proven themselves equipotent in previous studies [33, 34]. We speculated that the types of patients awaiting prostatectomy or biopsy contributed to the heterogen7eity, since the patients awaiting prostatectomy were all biopsy-proven. However, it was not identified in this study. The size of the lesions, primarily affecting the elastography results in deep structures [35], was not included in the sub-group analysis because relevant data is not provided in the papers.

MpUS is composed of multiple modalities, and there are great differences in the operation process and interpretation methods among different studies. Standardization is an inevitable trend in the development of mpUS. Unlike MRI, ultrasound relies heavily on the operator’s experience, so it is more difficult to standardize. How to establish a standardized diagnostic system is a problem that needs to be solved. Machine learning can identify a large number of complex imaging features, providing far more information than conventional methods [36–38]. In recent years, more and more attention has been paid to the advantages of artificial intelligence in imaging diagnosis. Several studies have preliminarily explored the application of artificial intelligence in mpUS, showing promising results [19, 39]. More relevant research is needed in the future.

There are several limitations in the present study. First, there are great differences between studies, which reduces the reliability of the pooled results. Therefore, we performed a subgroup analysis and found that the sensitivity and specificity of the diagnosis of csPCa were lower than that of any PCa, which might be one of the sources of heterogeneity. However, due to the limitation of the number of studies and the variable definitions of csPCa used in the included studies, the diagnostic performance of mpUS for csPCa is still not fully understood. Second, three of the papers all include patients who underwent mpUS with a known diagnosis of prostate cancer awaiting prostatectomy. This introduces significant bias, particularly given that the sonographer was aware of this when performing the prostate US. Third, according to existing studies, mpUS was defined as the combination of B-mode, elastography, and contrast-enhanced ultrasound. However, other ultrasound modalities also show promising results in the diagnosis of PCa, such as micro-US and HistoScanning [40]. Whether the addition of these modalities can increase the accuracy of mpUS in the diagnosis of PCa has not been reported yet. Fourth, as far as we know, this is the first meta-analysis focusing on the value of mpUS in the diagnosis of PCa, providing valuable information for urologists and sonographers. We found that mpUS had moderate accuracy in the diagnosis of PCa, but we could not compare the difference in the diagnostic accuracy between mpUS and mpMRI. More studies are needed in the future to directly compare mpUS and mpMRI head-to-head. Fifth, publication bias was found, and we only considered English-language studies, which may have impacted our results.

## Conclusion

MpUS is a valuable tool in the diagnosis of prostate cancer. It has moderate diagnostic accuracy, and the diagnostic accuracy for csPCa is significantly lower than any PCa. The standardized diagnosis system of mpUS is needed to be established in the future, and external validation is also necessary. The head-to-head comparison between mpUS and mpMRI is also a focus of future research.

### Supplementary Information

**Additional file 1.** Strategy for PubMed, Embase, Cochrane Library, Scopus, and Web of Science. Strategy for ClinicalTrials.gov. Strategy for Google Scholar.

## Data Availability

All data generated or analyzed during this study are included in this published article and its additional files.

## Abbreviations

CI: Confidence interval
csPCa: Clinically significant prostate cancer
mpMRI: Multiparametric magnetic resonance imaging
mpUS: Multiparametric ultrasound
PCa: Prostate cancer
SROC: Summary receiver operating characteristic
US: Ultrasound
