# Supplementary material for: Diagnostic accuracy of multiparametric ultrasound in the diagnosis of prostate cancer: systematic review and meta-analysis
Source: Insights Imaging. 2023 Nov 24;14:203. doi: 10.1186/s13244-023-01543-1 (PMC10673798; doi:10.1186/s13244-023-01543-1)
Supplement: Supplementary file 1 — Additional file 1. Strategy for PubMed, Embase, Cochrane Library, Scopus, and Web of Science. Strategy for ClinicalTrials.gov. Strategy for Google Scholar. [file 13244_2023_1543_MOESM1_ESM.docx]

**Diagnostic accuracy of multiparametric ultrasound in the diagnosis of prostate cancer: systematic review and meta-analysis**

**ELECTRONIC SUPPLEMENTARY MATERIAL**

**Strategy for PubMed, Embase, Cochrane Library, Scopus, Web of Science:**

#1 (contrast-enhanced[Title/Abstract]) OR (contrast enhanced[Title/Abstract])

#2 (Elasticity Imaging Techniques[Title/Abstract]) OR (Elasticity Imaging Technique[Title/Abstract])) OR (Imaging Technique, Elasticity[Title/Abstract])) OR (Imaging Techniques, Elasticity[Title/Abstract])) OR (Technique, Elasticity Imaging[Title/Abstract])) OR (Techniques, Elasticity Imaging[Title/Abstract])) OR (Tissue Elasticity Imaging[Title/Abstract])) OR (Elasticity Imaging, Tissue[Title/Abstract])) OR (Elasticity Imagings, Tissue[Title/Abstract])) OR (Imaging, Tissue Elasticity[Title/Abstract])) OR (Imagings, Tissue Elasticity[Title/Abstract])) OR (Tissue Elasticity Imagings[Title/Abstract])) OR (Elastography[Title/Abstract])) OR (Elastographies[Title/Abstract])) OR (Vibro-Acoustography[Title/Abstract])) OR (Vibro Acoustography[Title/Abstract])) OR (Vibro-Acoustographies[Title/Abstract])) OR (Sonoelastography[Title/Abstract])) OR (Sonoelastographies[Title/Abstract])) OR (Acoustic Radiation Force Impulse Imaging[Title/Abstract])) OR (ARFI Imaging[Title/Abstract])) OR (ARFI Imagings[Title/Abstract])) OR (Imaging, ARFI[Title/Abstract])) OR (Imagings, ARFI[Title/Abstract])) OR (Elastograms[Title/Abstract])) OR (Elastogram[Title/Abstract])

#3: #1 and #2

#4 (Multiparametric Ultrasonography[Title/Abstract]) OR (Multiparametric Ultrasound[Title/Abstract])) OR (Multiparametric transrectal Ultrasound[Title/Abstract])) OR (Multiparametric transrectal Ultrasonography[Title/Abstract])) OR (Multiparametric transperineal Ultrasonography[Title/Abstract])) OR (Multiparametric transperineal Ultrasound[Title/Abstract])

#5 #3 or #4

#6 (Prostatic Neoplasms[Title/Abstract]) OR (Prostate Neoplasms[Title/Abstract])) OR (Neoplasms, Prostate[Title/Abstract])) OR (Neoplasm, Prostate[Title/Abstract])) OR (Prostate Neoplasm[Title/Abstract])) OR (Neoplasms, Prostatic[Title/Abstract])) OR (Neoplasm, Prostatic[Title/Abstract])) OR (Prostatic Neoplasm[Title/Abstract])) OR (Prostate Cancer[Title/Abstract])) OR (Cancer, Prostate[Title/Abstract])) OR (Cancers, Prostate[Title/Abstract])) OR (Prostate Cancers[Title/Abstract])) OR (Cancer of the Prostate[Title/Abstract])) OR (Prostatic Cancer[Title/Abstract])) OR (Cancer, Prostatic[Title/Abstract])) OR (Cancers, Prostatic[Title/Abstract])) OR (Prostatic Cancers[Title/Abstract])) OR (Cancer of Prostate[Title/Abstract])

#7 #5 and #6

**Strategy for ClinicalTrials.gov:**

Condition or disease :[prostate]

Synonyms of conditions or disease (4): prostate; Prostatic; PROSTATE GLAND; Prostates; Prostatic Gland

Other terms :[Ultrasound] OR [Ultrasonography]

Study Results: With results

**Strategy for Google Scholar:**

#1 intitle:"prostate cancer" intitle:"Multiparametric Ultrasonography"

#2 intitle:"prostate cancer" intitle:"Multiparametric Ultrasound"

#3 intitle:"prostate cancer" intitle:"Multiparametric transrectal Ultrasound"

#4 intitle:"prostate cancer" intitle:"Multiparametric transperineal Ultrasonography"

#5 intitle:"prostate cancer" intitle:"Multiparametric transrectal Ultrasonography"

#6 intitle:"prostate cancer" intitle:"Multiparametric transperineal Ultrasound"

#7 #1 or #2 or #3 or #4 or #5 or #6
